# Transcatheter arterial embolization followed by laparoscopic anatomic hepatectomy for spontaneous rupture of a giant hepatic angiomyolipoma: a case report

**DOI:** 10.3389/fsurg.2023.1329535

**Published:** 2023-12-21

**Authors:** Jianjun Wang, Ruizi Shi, Hua Luo, Pei Yang, Huiwen Luo, Ziqing Gao, Decai Wang, Xintao Zeng

**Affiliations:** ^1^ Department of Hepatobiliary Surgery, Mianyang Central Hospital, School of Medicine, University of Electronic Science and Technology of China, Mianyang, China; ^2^ NHC Key Laboratory of Nuclear Technology Medical Transformation, Mianyang Central Hospital, School of Medicine, University of Electronic Science and Technology of China, Mianyang, China; ^3^ Department of Pathology, Mianyang Central Hospital, School of Medicine, University of Electronic Science and Technology of China, Mianyang, China; ^4^ Department of Urology, Mianyang Central Hospital, School of Medicine, University of Electronic Science and Technology of China, Mianyang, China

**Keywords:** case report, hepatic angiomyolipoma, rupture, transcatheter arterial embolization, laparoscopic hepatectomy

## Abstract

Hepatic angiomyolipoma is a rare and possibly cancerous mesenchymal tumor that consists of three components: blood vessels, smooth muscle cells, and adipose tissue. In this paper, we reported a case of a 36-year-old man who had a giant hepatic angiomyolipoma with spontaneous rupture and hemorrhage. The patient was admitted to our hospital with sudden upper abdominal pain for 3 h. A giant tumor was found in the left and caudate lobes of the liver, as well as significant blood collection around the liver and in the pelvis. Hemoglobin, liver function test results, and serum tumor maker levels were all within normal ranges. To prevent bleeding, emergency angiography and embolization were performed. During angiography, it was discovered that the tumor was supplied by the left hepatic artery and had a very rich internal blood supply. A massive left hepatic mass of about 11 cm in diameter was found bulging from the surface of the liver and rupturing there during laparoscopic exploration a week later. The rupture was strongly adhered to the smaller curvature of the stomach. The patient underwent laparoscopic left hemihepatectomy and caudate lobectomy, and the tumor specimen was brown, with clear boundaries with the surrounding normal liver parenchyma, and there were a large number of necrotic lesions inside the tumor. Histopathological results confirmed the mass as hepatic angiomyolipoma with negative resection margins. Immunohistochemical staining indicated that the tumor had positive homatropine methylbromide-45. After 13 months of follow-up, no tumor recurrence or metastasis occurred in the patient.

## 1. Introduction

Angiomyolipoma (AML) is a rare tumor that originates from mesenchymal tissue and contains varying amounts of adipose tissue, smooth muscle cells, and proliferating blood vessels (1). The liver ranks as the second most frequent site of AML involvement after the kidney (1). Tuberous sclerosis may be associated with certain instances of hepatic angiomyolipoma (HAML) (2).

Due to the lack of typically identifiable symptoms, HAML diagnosis is usually an accidental discovery on abdominal imaging. HAML is a rare liver tumor, with spontaneous rupture and hemorrhage being even rarer. According to a research published in 2021, a total of 10 HAML patients have experienced spontaneous rupture so far (3). In renal AML, spontaneous rupture and hemorrhage can be a presenting symptom, with lesions larger than 4 cm being a risk factor (4). However, the limited number of reported spontaneous rupture cases of HAML prevents the identification of risk factors for liver rupture. Despite numerous reports of HAML cases in the English literature, most focus on differential diagnosis of incidentally detected solid liver lesions. The diagnosis of HAML is challenging on imaging because of the different proportions of blood vessels, smooth muscle cells, and adipose tissue. In this study, we reported a rare case of a 36-year-old man with a giant HAML with spontaneous rupture and hemorrhage, and imaging showed the presence of large amounts of fat, which led us to consider HAML rather than hepatocellular carcinoma (HCC) or other common liver tumors at the initial diagnosis.

## 2. Case report

**Figure 1:**
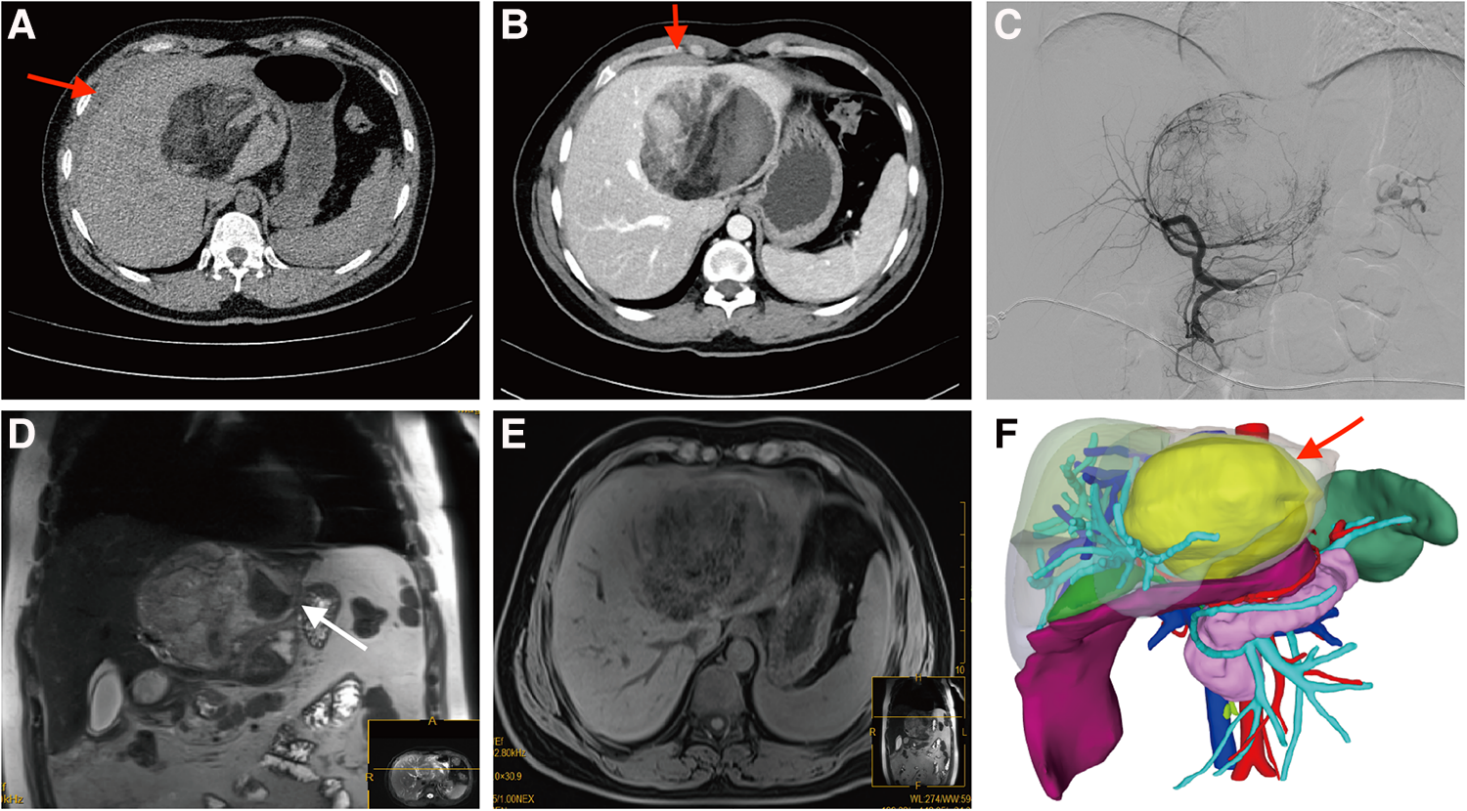
Preoperative imaging findings. (**A,B**) Abdominal computed tomography (CT) scans revealed a prominently sized mass with heterogeneous density located within the left hepatic lobe. In addition, intraabdominal bleeding (red arrow) was observed near the liver. The contrast-enhanced CT scan revealed heterogeneous enhancement within the lesion, which showed close adherence to the middle hepatic vein and the curvature of the stomach. (**C**) Angiography findings showed significant thickening and increased branching of the left hepatic artery, suggesting a robust blood supply to the tumor. (**D,E**) The hypointense signal observed on the MRI confirmed that the lesion was not consistent with a hepatocellular tumor. The white arrow indicated the rupture of the tumor. (**F**) 3D reconstruction model based on CT results. The purple area below the liver indicated blood accumulation. The yellow arrow indicated the tumor.

A 36-year-old male was admitted to our hospital with sudden upper abdominal pain for 3 h. Abdominal palpation revealed upper abdominal tenderness. The patient and his family members had no history of hepatitis, cirrhosis, or alcoholism. His body temperature was 36.6°C, heart rate 68 beats/min, respiratory rate 18 breaths/min and blood pressure 131/82 mmHg. Hemoglobin level was 122 g/L, prothrombin time 16 s, albumin 52.7 g/L, aspart aminotransferase 23 U/L, and alanine aminotransferase 39 U/L. The patient's common serum tumor markers [such as alpha-fetoprotein (AFP), PIVKA-II, carcinoembryonic antigen, carbohydrate antigen 125, and carbohydrate antigen 19-9] were all within normal limits. Abdominal enhanced computed tomography (CT) revealed that a 11 cm × 9 cm mass was in the left lobe and caudate lobe of the liver, with some components exhibiting fat density (Figure 1). The mass was located near the middle hepatic vein. Enhanced scans revealed an inhomogeneous enhancement of the mass, with blood vessels visible within the lesion. Abdominal ultrasound revealed blood accumulation around the liver and in the pelvis. We punctured the patient's left femoral artery using the Seldinger technique under color Doppler ultrasound guidance, then placed a 5F catheter, and finally performed hepatic and abdominal arteriography. The angiography revealed that the left hepatic artery was thickened with more branches, suggesting that the tumor was supplied by this artery. To prevent further bleeding, we embolized the patient's left hepatic artery with polyvinyl alcohol microspheres.

**Figure 2:**
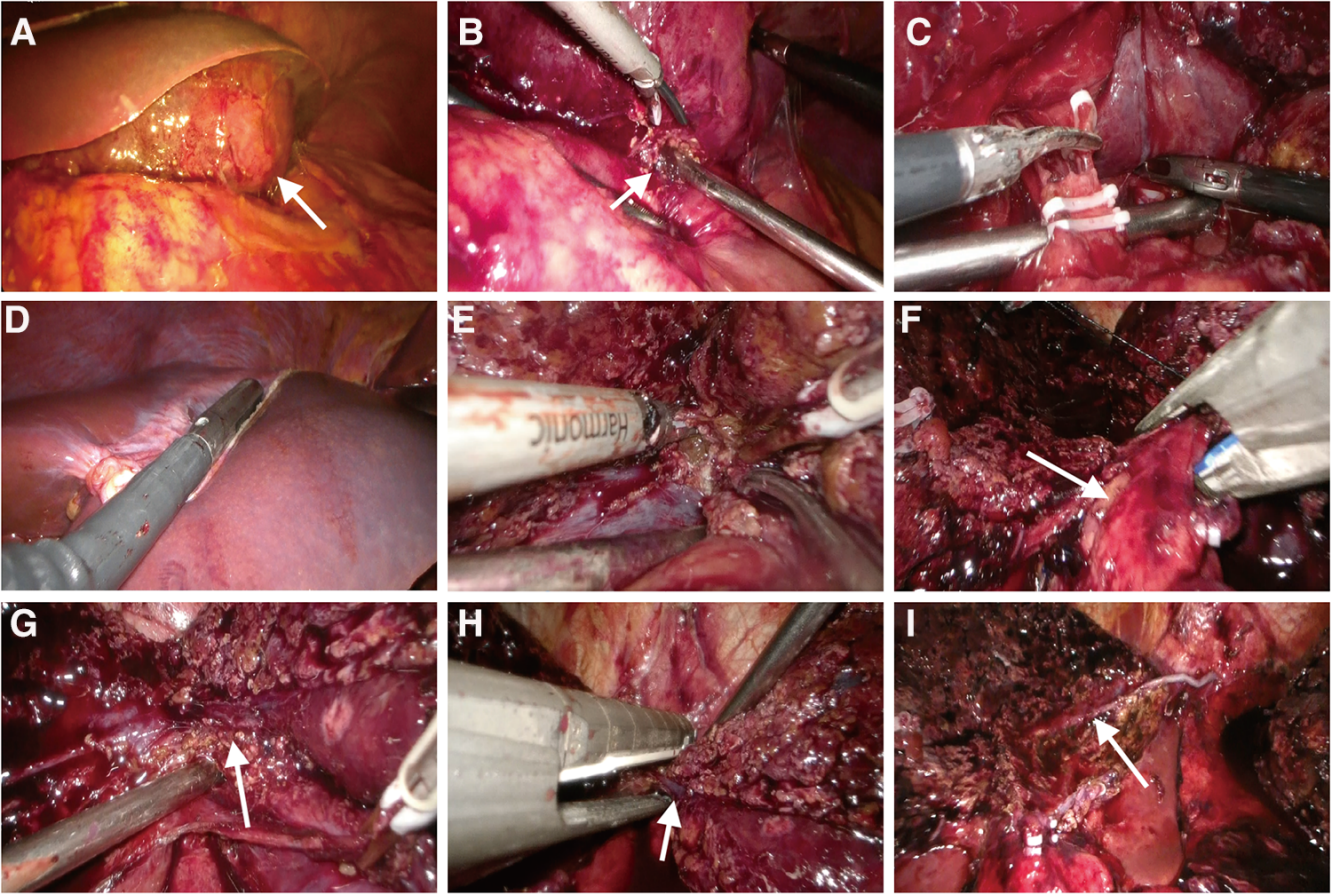
Intraoperative images. (**A**) Intraoperative exploration revealed a large mass (white arrow) in the left lobe of the liver. The hepatic parenchyma around the mass was normal, and the mass was severely attached to the stomach. (**B**) The rupture (white arrow) of the tumor was strongly adhered to the smaller curvature of the stomach. (**C**) There was noticeable hyperemia and edema of the hepatoduodenal ligament, together with obvious adhesions. The left hepatic artery was severed, and due to unclear hepatic portal structure, no further intrathecal separation of the left portal vein was performed. (**D**) Intraoperative ultrasound was used to determine tumor boundaries. (**E**) The hepatic parenchyma was transected along the middle hepatic vein. (**F**) Detachment of the left hepatic pedicle (white arrow). (**G**) The confluence of left and right hepatic veins (white arrow) was exposed. (**H**) The left hepatic vein (white arrow) was transected by ENDO-GIA. (**I**) Cross-sectional view of the liver after mass resection. The white arrow indicated the middle hepatic vein.

**Figure 3:**
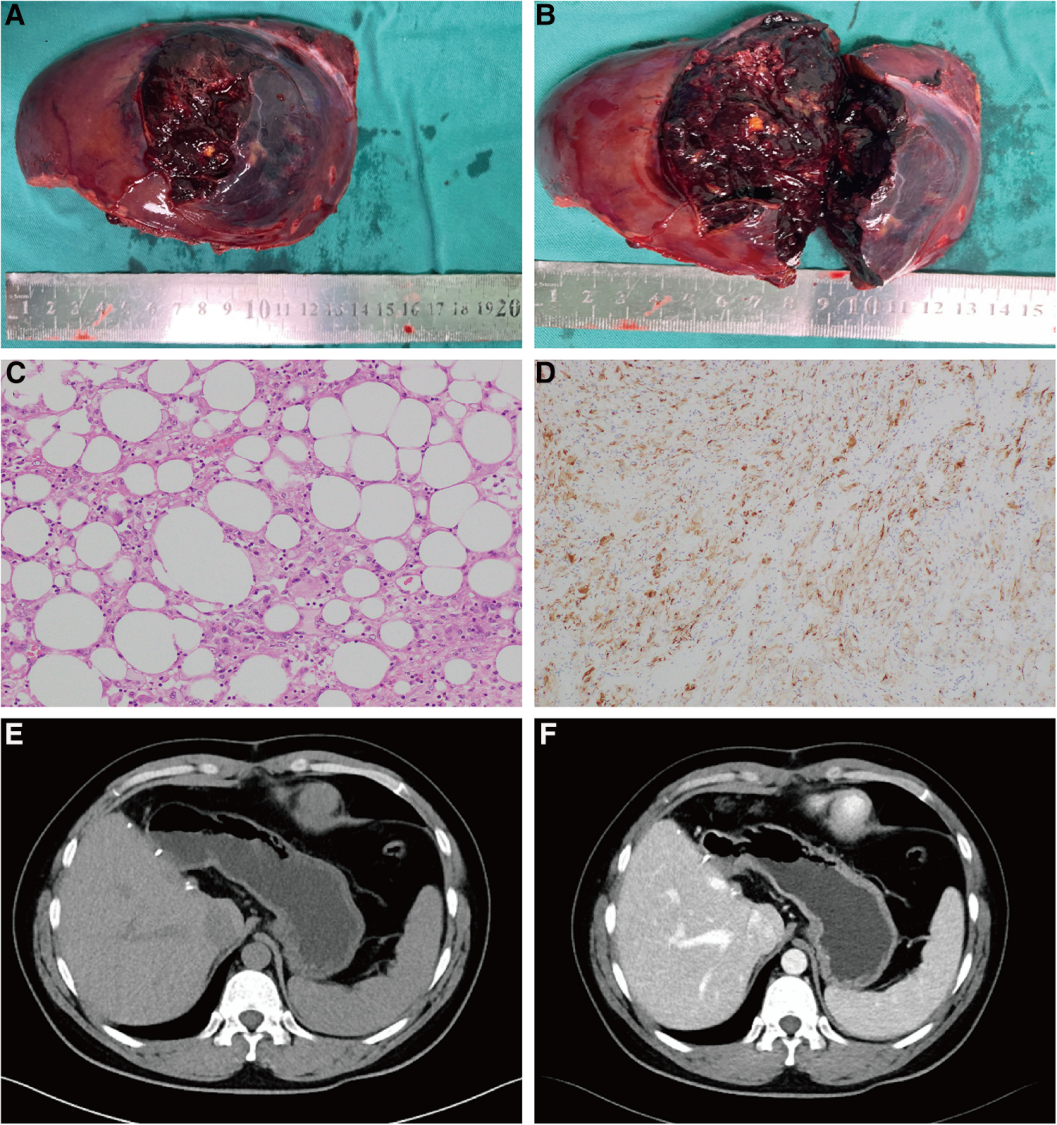
Postoperative histopathological results. (**A**) The tumor measured approximately 11 cm in diameter, exhibited a brown hue, and presented a smooth surface. (**B**) There was obvious hemorrhage and necrotic tissue within the tumor. (**C**) The tumor contained numerous adipocytes and epithelioid cells (HE staining; magnification × 200). (**D**) Tumor cells were positive for HMB-45 (immunohistochemical staining, magnification × 100). (**E,F**) One year postoperative CT follow-up results. No tumor recurrence or metastasis was detected in the patient.

The imaging presentation of HAML varies significantly, mainly influenced by the proportion of fat, smooth muscle, and vascular elements. And the key to HAML diagnosis depends on the amount of fat present (5). Abdominal CT revealed fat density and blood vessels in the mass and magnetic resonance imaging (MRI) on day 5 after the interventional embolization procedure revealed that the tumor signal was hyperintense in T2-weighted sequences and variable in T1-weighted sequences. The opposed-phase sequences revealed a drop-out tumor signal, indicating the presence of fat in the mass, further confirming that it was likely HAML. In order to facilitate the patient's swift recovery, we chose laparoscopic surgery rather than open surgery. The patient underwent laparoscopic surgery on day 7 following interventional embolization (Figure 2). Laparoscopic examination revealed an 11 cm diameter tumor in the left and caudate lobe of the liver, protruding from the liver surface and rupturing locally, with the rupture immediately adhering to the smaller curvature of the stomach. Subsequently, the patient underwent laparoscopic left hemihepatectomy and caudate lobectomy. The tumor specimen is brown in color and has a clear boundary with surrounding normal liver parenchyma tissue. He was discharged from the hospital on day 5 after a good postoperative recovery. Histological examination showed that the tumor was composed of proliferating blood vessels, smooth muscle cells, and adipose tissue, and the resection margins of the mass were negative (Figure 3). Immunohistochemistry staining indicated that the tumor had positive homatropine methylbromide-45 (HMB-45), melan-A, smooth muscle actin (SMA), CD34 but was negative for S100 protein. The Ki-67 index was 6%. Therefore, the final diagnosis of this patient was considered to be HAML.

## 3. Discussion

HAML, first described in 1976, is a rare solid mesenchymal tumor that typically occurs in the non-cirrhotic liver and predominantly affects middle-aged women (6). Approximately 60% of HAML cases are found primarily in the right liver, with 84% of cases presenting only one lesion (1, 7). HAML is often asymptomatic, with most patients (42%–72%) discovering the tumor during routine physical examinations (8). In 2021, Calame et al. reported that a total of 10 HAML patients experienced spontaneous rupture (3). In renal AML, pregnancy and tumor size (≥4 cm) are two recognized risk factors for susceptibility to rupture (9). However, the limited number of reported spontaneous rupture cases of HAML to date prevents the identification of risk factors for liver rupture. Table 1 provides a comprehensive summary of the cases of spontaneous rupture of HAML. In previous studies, spontaneously ruptured HAML was usually small (median 4 cm) and only one case of HAML with a size greater than 10 cm (19). In our study, the HAML measured approximately 11 cm in size and was situated close to the middle hepatic vein. Emergency transcatheter arterial embolization was performed to prevent further bleeding, followed by laparoscopic hepatectomy a week later. The patient has been monitored for 13 months and shows no signs of tumor recurrence or metastasis (Figure 3).

**Table 1:** Cases of spontaneous rupture of HAML.

Ref.	Age, yr	Sex	Symptoms	Diagnostic approaches	Size, cm	Location	Single or Multiple	Treatment	Outcomes
Huber et al. (10)	22	F	Hemorrhagic shock	CT	8	Segment III	Multiple	Surgical resection of segments II and III	The patient recovered well and was discharged on the 12th day after surgery
Guidi et al. (11)	74	M	Abdominal pain	CT	10	Segment I	Multiple	Surgical resection of the hemorrhagic liver mass	The patient recovered well and was discharged on the 8th day after surgery
Tsui et al. (12)	41	M	NM	NM	9	Right lobe of liver	Single	Surgical resection	Patient in healthy condition 4 year after surgery
Zhou et al. (13)	NM	NM	Hemorrhagic shock	Ultrasonography	5	Right lobe of liver	Single	Laparotomy for hemostasis	No tumor recurrence or metastasis was found during follow-up
Ding et al. (14)	56	F	Shock	Emergent laparotomy	6	Segment IV	Single	Segmental resection of the liver	No serious postoperative complications
Occhionorelli et al. (15)	25	F	Abdominal pain and hypotension	CT	9	Left lobe of liver	Single	After 48 h of manual compression, left hepatic lobectomy was performed	The patient recovered well and was discharged on the 9th day after surgery
Aoki et al. (16)	70	M	Back pain on the right side	CT	7	Segment VII	Single	Transcatheter arterial embolization, right lobectomy of the liver 39 days later	Five days postoperatively, she developed thrombus in her left popliteal vein and left pulmonary artery. Insertion of an IVC filter which was removed due to sepsis. She was discharged 24 days after surgery
Tajima et al. (17)	38	M	Abdominal pain	CT	10.5	Right lobe of liver	Single	Transcatheter arterial embolization and right lobectomy of the liver	Not mentioned
Kai et al. (18)	77	F	Abdominal pain and transient loss of consciousness	CT	2.3	Segment II	Single	Conservative initial treatment with periodic imaging studies and laparoscopic left lateral segmentectomy was performed 4 months later	The patient recovered well and was discharged on the 7th day after surgery. No sign of recurrence at 3.5 years of follow-up
Kim et al. (19)	31	M	Severe abdominal pain	CT	12	Right lobe of liver	Single	Emergent angiography with embolization. Hepatic resection was performed 15 days later	The patient recovered well
Wu et al. (6)	49	F	Nausea and abdominal distention	CT	15	Left lobe of liver	Single	Surgical resection of the tumor	The patient recovered well and was discharged on the 6th day after surgery

HAML, hepatic angiomyolipoma; F, female; M, male; NM, not mentioned; CT, computed tomography; IVC, inferior vena cava.

Accurate diagnosis of HAML is challenging on imaging and depends largely on histopathologic findings. Individuals diagnosed with HCC usually have a medical history of cirrhosis, alcohol abuse or hepatitis. Moreover, HCC patients commonly present with elevated levels of AFP, which are typically absent in patients with HAML. On enhanced CT, typical HCC exhibits an accelerated enhancement in the arterial phase and a reduced enhancement in the portal phase. The equilibrium phase displays a low density alteration compared to normal hepatic parenchyma, known as “rapid wash-in and wash-out.” Similarly, typical HCC can also exhibit “rapid wash-in and wash-out” enhancement on MRI. However, the imaging of HAML exhibits significant variation due to the varying proportions of blood vessels, smooth muscle cells, and adipose tissue. Only 28.2% of patients with HAML can be accurately diagnosed before surgery and histopathologic results are available (1). In our study, abdominal CT revealed fat density and blood vessels in the mass, leading to the initial diagnosis of HAML, and MRI showed that the tumor signal was drop-out on the opposed-phase sequences, so we initially diagnosed the patient with HAML rather than HCC or other common liver tumors.

Histopathologic and immunohistochemical examination is the gold standard for HAML diagnosis. Macroscopically, HAML tumors have a clear perimeter, no envelope, and a smooth, brownish surface; however, hemorrhage or intratumoral necrosis can alter the appearance. In this case, the tumor specimen appeared brown in appearance, with clear boundaries with surrounding normal tissues, and a large number of necrotic lesions inside the tumor. Microscopically, cells usually have a clear or slightly eosinophilic cytoplasm with small, concentrated, round or oval nuclei and small nucleoli (20). By immunohistochemistry, HAML is positive for melanocytic markers, such as HBM-45 and melan-A, with HBM-45 being the most specific and commonly used marker (21). Smooth muscle markers (e.g., actin and/or desmin) are variedly stained in HAML. In addition, epithelial markers, S100 protein, or AFP are not expressed in HAML (22).

Cases of spontaneous rupture and hemorrhage in HAML are rare, but there are relatively more cases of rupture and hemorrhage in other liver tumors, especially in HCC. For patients with hepatic tumors with unknown diagnosis and accompanied by rupture and bleeding, angiography combined with arterial embolization or emergency laparotomy are common treatment options, and the choice of treatment plan is mainly determined by the experience of each medical center. Some medical centers choose emergency surgery to resect the tumor to achieve hemostasis, while others choose interventional embolization to prevent bleeding. In this case, we chose to perform interventional embolization first for the following reasons: First, although we initially diagnosed the patient as HAML through enhanced CT results, we were still unable to confirm the diagnosis. To define the first diagnosis, additional auxiliary examinations, including MRI, may be performed after interventional embolization. Secondly, after interventional embolization, we had more time to evaluate the patient's condition, adjust the preoperative status of the patient, and formulate a detailed surgical plan, so as to achieve the goal of reducing postoperative complications and hospital stay. In this case, we could almost confirm that the patient was HAML through imaging results, and no metastatic lesions were found in other organs. After confirming that the remaining liver volume was sufficient by a three-dimensional model, we performed laparoscopic left hemihepatectomy and caudate lobectomy. During the surgery, we removed about 26% of the liver volume. The patient recovered well after surgery and was discharged on the fifth day after surgery. To date, the patient has been monitored for 13 months and has not experienced any discomfort.

HAML is usually considered a benign tumor; however, its aggressive potential is attracting increasing attention as evidence accumulates. Calame et al. summarized some of the risk factors that may be associated with recurrence or metastasis, including tumor size greater than 5 cm, infiltrative growth pattern, high nuclear grading, necrosis, and mitotic activity > 1/50 high power field (3). Therefore, surgery should be considered for suspected HAML patients, and those with risk factors for recurrence or metastasis require further monitoring even after radical resection for a better prognosis.

## Data Availability

The raw data supporting the conclusions of this article will be made available by the authors, without undue reservation.
